# Prevalence and determinants of inadequate dietary diversity among pregnant women in four Sub-Saharan Africa countries: a multilevel analysis of recent demographic and health surveys from 2021 to 2022

**DOI:** 10.3389/fnut.2024.1405102

**Published:** 2024-09-05

**Authors:** Alebachew Ferede Zegeye, Enyew Getaneh Mekonen, Tadesse Tarik Tamir, Belayneh Shetie Workneh

**Affiliations:** ^1^Department of Medical Nursing, School of Nursing, College of Medicine and Health Sciences, University of Gondar, Gondar, Ethiopia; ^2^Department of Surgical Nursing, School of Nursing, College of Medicine and Health Sciences, University of Gondar, Gondar, Ethiopia; ^3^Department of Pediatrics and Child Health Nursing, School of Nursing, College of Medicine and Health Sciences, University of Gondar, Gondar, Ethiopia; ^4^Department of Emergency and Critical Care Nursing, School of Nursing, College of Medicine and Health Sciences, University of Gondar, Gondar, Ethiopia

**Keywords:** determinants, inadequate dietary diversity, prevalence, pregnant women, Sub-Saharan Africa

## Abstract

**Background:**

Diversity in the mother’s diet can have major effects on the developing fetus throughout pregnancy. Approximately 1 million neonates die on their first day of life as a result of inadequate nutrition, which also complicates the mother’s pregnancy and has a negative impact on the delivery outcome. Dietary diversity during pregnancy is poorly recognized in developing countries, despite the fact that it is detrimental. As a result, this study aimed to assess the prevalence and associated factors of inadequate dietary diversity in Burkina Faso, Ghana, Kenya, and Tanzania among pregnant women.

**Methods:**

Secondary data analysis was conducted using data from the most recent Demographic and Health Surveys, which included four countries in Sub-Saharan Africa between 2021 and 2022. A total of 80,083 pregnant women were included in this study. The women’s dietary diversity was computed from 10 food categories. Based on the minimum diversity score, women were categorized as having inadequate dietary diversity if their diversity score was less than five food items and as having adequate dietary diversity if they took five or more food groups. A multilevel mixed-effects logistic regression model was used to identify the factors associated with inadequate dietary diversity. At *p*-values <0.05, significant factors correlated with inadequate diversity were identified. The result was interpreted using 95%CI and adjusted odds ratio. The best-fit model was determined to be the one with the lowest deviance and highest loglikelihood ratio.

**Results:**

The prevalence of inadequate dietary diversity among pregnant women in Burkina Faso, Ghana, Kenya, and Tanzania was 94.46%. Factors such as no formal education (AOR = 3.39, 95% CI: 2.54, 4.54), distance to health facilities (AOR = 1.36, 95% CI: 1.16, 1.60), poor wealth quantiles (AOR = 2.97, 95% CI: 2.41, 3.65), no media exposure (AOR = 1.84, 95% CI: 1.45, 2.35), low community ANC utilization (AOR = 1.21, 95% CI: 1.16, 1.60), and reside Burkina Faso (AOR = 1.47, 95% CI: 1.09, 1.99) were among the factors associated with inadequate dietary diversity.

**Conclusion:**

According to this study finding, pregnant women had a high percentage of inadequate dietary diversity. Factors at the individual and community levels contributed to the lack of diversity in nutrition. Thus, when developing policies and strategies, the health ministries of Burkina Faso, Kenya, Ghana, and Tanzania should to consider the women who underutilize antenatal care services, live in low wealth quantiles and who did not get formal education.

## Background

Eating foods from various food groups during pregnancy has been known as dietary diversity among pregnant women (1). It is a measure of the quality and variety of the diet, as well as the adequacy of micronutrients, which are essential for the health and development of the mother and the fetus (2, 3). To ensure that women enter pregnancy and breastfeeding without deficiencies, pregnant women need increased intake of protein, iron, iodine, vitamin A, foliate, fruits, vegetables, and animal products (4). Pregnant women who eat a diverse diet are less likely to experience intrauterine growth restriction (IUGR), abortion, preterm birth, low birth weight, maternal anemia, and other adverse birth outcomes (5–7). Moreover, it can enhance the immune systems, cognitive abilities, and mental health of both the mother and the child (2).

A World Bank report highlights that insufficient nutrition during pregnancy has lasting and significant consequences for both mothers and their children. Poor maternal and child nutrition can potentially reduce a country’s economic productivity by 2–3% annually (6, 8). Annually, developing countries bear nearly 99% of all maternal mortality due to inadequate nutrition. The primary cause of indirect maternal mortality is iron deficiency anemia, which puts pregnant women at risk of sepsis, bleeding, and death during childbirth (9–11). In Sub-Saharan Africa, pregnant women face a heightened risk of malnourishment due to inadequate micronutrient intake. This deficiency contributes to 7% of the global illness burden, at least 15% of maternal deaths, and adverse maternal outcomes (12, 13).

In low and middle-income countries, pregnant women often lack dietary diversity; only 20% in Pakistan, 25% in the Democratic Republic of the Congo (DRC), 50% in Guatemala, and 70% in India achieve adequate dietary variety (14). The previous studies found that inadequate dietary diversity was significantly associated with maternal education (15, 16), low income, large family size (5, 17, 18), lack of access to antenatal care, and nutrition counseling services (19).

Despite Sub-Saharan African countries sharing the huge burden of global maternal morbidity and mortality related to inadequate nutrition, the previous few studies focused at the institutional level, using a small sample size from primary data. However, this study analyzed factors at the individual and community levels that have a greater impact on inadequate dietary diversity among pregnant women. Therefore, the present study focused on investigating the prevalence and determinants of inadequate dietary diversity among pregnant women in four Sub-Saharan countries using a multilevel mixed effect analysis of the most recent Demographic and Health Survey from 2021 to 2022.

## Materials and methods

### Study setting

The portion of Africa south of the Sahara is known as the sub-Saharan region, and it is made up of four huge and diverse regions: Eastern Africa, Central Africa, Western Africa, and Southern Africa. The region encompasses an area of 9.4 million square miles, with an expected 407 million reproductive-age women by 2030 and 607 million by 2050 (20, 21).

Burkina Faso is a landlocked country in West Africa. It has a population of about 22.5 million. Pregnant women in Burkina Faso face high rates of nutritional iron deficiency (26%), which can lead to maternal mortality and poor birth outcomes (22). Ghana is a country in West Africa that borders the Gulf of Guinea and the Atlantic Ocean. It has a population of about 34.2 million. Pregnant women in Ghana also suffer from nutritional iron deficiency (26%), as well as low dietary diversity and intake of micronutrients (23).

Tanzania is a country in East Africa that lies within the African Great Lakes region. It has a population of about 61.7 million. Pregnant women in Tanzania face a double burden of malnutrition, with both under-nutrition and over-nutrition affecting their health and pregnancy outcomes (24). Kenya is a country in East Africa that borders the Indian Ocean and Lake Victoria. It has a population of about 51.5 million. Pregnant women in Kenya face similar challenges as those in Tanzania, with a high prevalence micronutrient deficiencies (25). This study was conducted based on the recent DHS survey data from four Sub-Saharan African countries, such as Burkina Faso, Ghana, Kenya, and Tanzania.

### Study design and period

The study employed a cross-sectional design, utilizing data from the Demographic and Health Surveys (DHS) conducted in four Sub-Saharan African countries. The DHS program collects household and individual data from a nationally representative sample, which includes information on health, nutrition, and population across various stages. For the purpose of this multilevel analysis, we focused on the DHS surveys of 2021 and 2022. The DHS employs a two-stage stratified sampling technique, which is ideal for multilevel modeling as it allows for the examination of both individual and community-level factors.

### Population and eligibility criteria

Pregnant women who are 15–59 years old in Burkina Faso, Ghana, Kenya, and Tanzania were the source population. The study population was all the pregnant women who were in the selected enumeration areas included in the analysis.

### Data source and sampling procedure

To gain insight into inadequate dietary diversity among pregnant women in four Sub-Saharan Africa countries and determinates, four sub-Saharan countries’ such as Burkina Faso, Ghana, Kenya, and Tanzania DHS surveys were appended together since other DHS data sets other than the above countries lack food indicators to determine dietary diversity. Previous studies justify the use of different survey years based on contextual factors and the need for robust evidence (19, 26–28). Several data-sets, including data on key health indicators like fertility, mortality, nutrition, maternal and child health, HIV/AIDS, and gender-based violence, are included in each country’s survey. Using a stratified two-stage cluster design, the Demographic and Health Survey first generates the enumeration areas and then creates a sample of households from each enumeration area in the second stage. The dependent and independent variables for each country were extracted from the individual record dataset (IR file) for this study and the data was subsequently appended using STATA/SE. The outcome variable (inadequate dietary diversity) was recoded as inadequate and adequate from the individual record (IR) data-set. The total number of diverse food groups that the pregnant women consumed in the 24 h before to the assessment was determined using the minimum dietary diversity score (MDD-W).

The women’s dietary diversity score (MDDW-10) for the 10-food categories served as the basis for the MDD-W indicator. These dietary groups include staples high in starch (grains, white roots and tubers, and plantains); fruits and vegetables high in vitamin A; dark green leafy vegetables; other vegetables; fruits; meat and fish; poultry; eggs; pulses/legumes; nuts and seeds; dairy products; and flesh foods (meat, fish, and poultry). MDD-W is predicted on a 24-h food recall period (29). Total weighted samples of 80,083 pregnant women were included in this study (Table 1).

**Table 1:** Sample size for inadequate dietary diversity and its determinants among pregnant women in four Sub-Saharan African countries, DHS 2021–2022.

Countries	Year of survey	Weighted sample (n)	Weighted sample (%)
Burkina Faso	2021	17,659	22.05
Ghana	2022	15,014	18.75
Kenya	2022	32,156	40.15
Tanzania	2022	15,254	19.05
Total weighted sample size		80,083	100

### Study variables

#### Dependent variable

The outcome of this study was inadequate dietary diversity, which was derived from the individual record (IR) data set. The women’s dietary diversity was computed from 10 food categories. These dietary groups include staples high in starch (grains, white roots and tubers, and plantains); fruits and vegetables high in vitamin A; dark green leafy vegetables; other vegetables; fruits; meat and fish; poultry; eggs; pulses/legumes; nuts and seeds; dairy products; and flesh foods (meat, fish, and poultry). Based on the minimum diversity score, the dietary diversity was then divided into two categories. Women were categorized as having inadequate dietary diversity if their diversity score was less than five food items and as having adequate dietary diversity if they took five or more food groups (30).

#### Independent variables

Independent variables from two sources (variables at the individual and community levels) were taken into account for this analysis because DHS data are hierarchical in nature.

**Level 1 or individual-level independent variables**: maternal age (6, 14–47), maternal educational status (no formal education, primary, secondary, higher), husband educational status (no formal education, primary, secondary, higher), maternal employment (not working, working), religion (orthodox, catholic, protestant, muslim, others), marital status of the mother (unmarried, married, ever married), number of ANC visits (no visit, 1–3, ≥4), total children ever born (≤3, 4–6, 7–9, >9), household wealth index (poor, middle, rich), distance to health facility (big problem, not big problem), household media exposure (no, yes), preceding birth interval (≤24 months, >24 months).

**Figure 1:**
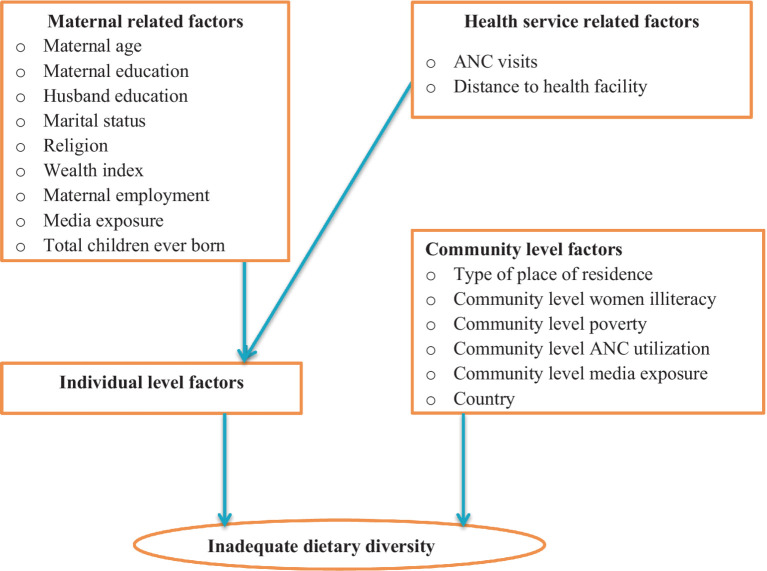
Conceptual framework for factors associated with inadequate dietary diversity among pregnant women in four Sub-Saharan African countries.

**Level 2 or community-level independent variables**: place of residence (urban or rural), community-level women’s illiteracy (low, high), community-level poverty (low, high), community-level media exposure (low, high), and community-level ANC utilization (low, high) country (Burkina Faso, Ghana, Kenya, Tanzania; Figure 1).

### Operational definition

#### Wealth index

The Wealth index in the Demographic and Health Surveys (DHS) is a measure that attempts to assess a household’s relative economic status using data on household assets, services, and amenities. It is constructed from existing survey data to examine various societal indicators, such as health, nutrition, education, and population, in relation to economic status (31).

#### Community-level women illiteracy

The percentage of women with at least a primary education is determined using data on respondents’ educational level. The individual level of women’s education was cross-tabulated using the cluster number (v001) and then classified using the national mean value. The communities with ≥50% of the national mean value of women’s education were classified as low community-level women’s illiteracy, while the communities with <50% of the national mean value of women’s community illiteracy were classified as high community-level women’s illiteracy (32).

#### Community-level poverty

The percentage of women in the rich and middle-class categories is taken into consideration when producing it. Following the computation of the cross-tabulating individual-level combined wealth index with the cluster number (v001), it was then categorized using the national mean value of the wealth index: low community-level poverty (communities with ≥50% of the national mean value of the community wealth index) and high community-level poverty (communities with <50% of the national mean value of the community wealth index) (33).

### Data processing and statistical analysis

After the data was extracted from recent DHS data sets, it was cleaned, entered, and analyzed with STATA/SE version 17 statistical software. In order to restore the representativeness of the survey and take the sample design into consideration when computing standard errors to generate correct statistical estimations, the data were weighted using the sampling weight, primary sampling unit, and stratum prior to doing any statistical analysis. To make the analysis survey-specific, we employed the weighting variable (v005) as a relative weight normalized. For the pooled data, we denormalized the individual standard weight of pregnant women by dividing it by the sample proportion of each country: [weight adjusted for women = V005× (total number of pregnant women in the entire country between the ages of 15 and 59 at the time of the survey)/(number of pregnant women aged 15–59 years in the survey)].

Since the DHS data are hierarchical, the usual logistic regression model’s assumptions about independence observations and equal variance are violated. When pregnant women are nested within a cluster and we assume that study subjects in the same cluster may have similar characteristics with participants in another cluster, we violate the independent observations and equal variance assumptions between clusters of the ordinal logistic regression model. This suggests that an advanced model that takes into account between-cluster factors is required. Given this, factors associated with inadequate dietary diversity have been identified using multilevel mixed-effects logistic regression. A binary logistic regression model was used to determine the factors associated with inadequate dietary diversity. Determinants of inadequate dietary diversity were reported in terms of an adjusted odds ratio (AOR) with a significance level of (95%). In the univariable analysis, at 95% confidence intervals with a *p*-value of <0.25 was considered a candidate for the multivariable analysis of data. All variables with *p* values <0.05 were considered statistically significant in multivariable logistic regression.

### Random effects

Random effects or measures of variation, such as the Likelihood Ratio test (LR), Intra-class Correlation Coefficient (ICC), and Median Odds Ratio (MOR), were taken into consideration in order to assess the variation in inadequate dietary diversity among women between clusters. Using clusters as a random variable, the ICC quantifies the degree of heterogeneity of inadequate dietary diversity between clusters, or the percentage of the total observed variation in inadequate dietary diversity that can be attributable to differences across clusters. This can be computed using the formula below: $ICC=\frac{VC}{VC+3.29}\times 100\%$ (34).

The median value of the odds ratio between the cluster at high likelihood of inadequate dietary diversity and the cluster at lower risk when individuals are randomly selected from two clusters is known as the Median Odds Ratio (MOR), which quantifies the variation or heterogeneity in inadequate dietary diversity between clusters in terms of odds ratio: MOR= *e*^0.95√VC^ (35).

Additionally, the PCV shows how variations in inadequate dietary diversity are explained by determinants and calculated as; $PCV=\frac{\text{Vnull}-Vc}{\text{Vnull}}\times 100\%$; where Vnull = variance of the null model and VC = cluster level variance (36). The association between the likelihood of inadequate dietary diversity and independent variables at the individual and community levels was estimated using fixed effects. With a *p*-value of less than 0.05, the adjusted odds ratio (AOR) and 95% confidence intervals were used to evaluate it and show its strength. Because the data are nested in nature, deviance = −2 (log likelihood ratio) and log likelihood ratio were used to compare the models; the model with the lowest deviance and the highest log likelihood ratio was selected as the best-fit model. By calculating the variance inflation factors (VIF), the multi-collinearity was assessed. As a result marital status and religion were avoided due to the variance inflation factor was beyond the acceptable rage (10).

### Model building for multilevel analysis

Multilevel mixed effect logistic regression uses four models: the null model (outcome variable only), model I (only individual level variables), model II (only community level variables), and model III (both individual and community level variables). The variation in inadequate dietary diversity within cluster was investigated using the null model, which lacks independent factors. The associations between the individual level factors and the outcome variable (Model I) and the association of community level variables with the outcome variable (Model II) were analyzed. In the final model (Model III), the association between the variables at the community and individual levels and the outcome variable was fitted simultaneously.


$$\log\left(\frac{\pi ij}{1-\pi ij}\right)\beta o+\beta 1ij+\beta 2xij+\ldots uj+eij$$

The likelihood of inadequate dietary diversity intake usage is represented by πij, whereas the probability of adequate dietary diversity intake use is represented by 1 − πij. When the effect of every explanatory variable is absent, the intercept, or ß0, represents the effect of feeding inadequate dietary diversity. For the ith individual in group j, the individual and community-level variables are denoted by β1xij, respectively. Since the ß’s are fixed coefficients, an increase in X can result in an increase in the likelihood of ß units, feeding inadequate dietary diversity. For the j^th^ clusters, the uj indicates the random effect, that is, the influence of the community and/or clusters on the mother’s choice for an inadequate dietary diversity (37, 38).

## Results

### Socio-demographic and economic characteristics of pregnant women in four Sub-Saharan African countries, DHS 2021–2022

A total of 80,083 pregnant women from Burkina Faso, Ghana, Kenya, and Tanzania were included in this study. A quarter of women (24.77%) had no formal education. Nearly one-third (28.81%) of pregnant women had a big problem to access healthcare institutions due to distance, and about 31,416 (39.23%) were living in rural areas of Sub-Saharan Africa. About 1.47% of pregnant women did not have ANC visits. More than half (50.6%) of pregnant women had low access to media (Table 2).

**Table 2:** Socio-demographic and economic characteristics of pregnant women in four Sub-Saharan African countries, DHS 2021–2022.

Variables	Frequency (n)	Percent (%)
**Individual level factors**		
Maternal age		
15–19	30,582	38.19
20–35	24,365	30.42
36–49	25,136	31.39
Maternal educational level		
No formal education	19,834	24.77
Primary	23,980	29.94
Secondary	29,528	36.87
Higher	6,741	8.42
Husband educational level		
No formal education	47,169	58.9
Primary	14,406	17.99
Secondary	13,074	16.33
Higher	5,434	6.79
Maternal occupational status		
Not working	31,076	38.8
Working	49,007	61.2
Marital status of the mother		
Never married	29.15	29.15
Currently married	49,142	61.36
Formerly/ever married	7,599	9.49
Distance to health facility		
Big problem	18,676	28.81
Not a big problem	46,152	71.19
Number of ANC visits		
No visit	1,175	1.47
1–3	7,186	8.97
≥4	71,722	89.56
Total children ever born		
≤3	55,859	69.75
4–6	18,125	22.63
7–9	5,282	6.6
>9	817	1.02
Preceding birth interval		
≤24 months	7,504	16.47
>24 months	38,065	83.53
Household wealth index		
Poor	30,670	38.3
Middle	16,029	20.02
Rich	33,384	41.69
Household media exposure		
No	18,047	22.54
Yes	62,036	77.46
**Community level factors**		
Place of residence		
Rural	31,416	39.23
Urban	48,667	60.77
Community media exposure		
Low	40,524	50.6
High	39,559	49.4
Community poverty		
Low	39,029	48.74
High	41,054	51.26
Community women’s’ literacy		
Low	42,402	52.95
High	37,681	47.05
Community ANC utilization		
Low	30,431	38.02
High	49,617	61.98
Country		
Burkina Faso	17,659	22.05
Ghana	15,014	18.75
Kenya	32,156	40.15
Tanzania	15,254	19.05

### Magnitude of inadequate dietary diversity among pregnant women in four Sub-Saharan African countries

**Figure 2:**
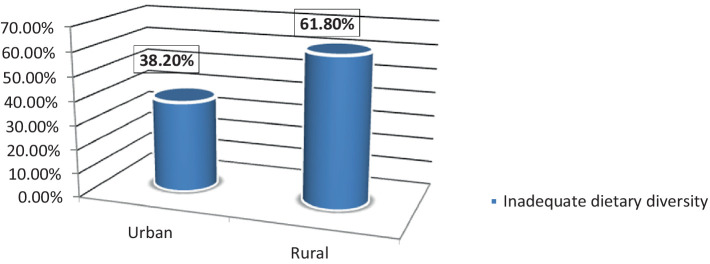
Urban and rural prevalence of inadequate dietary diversity among pregnant women in four Sub-Saharan African countries.

**Figure 3:**
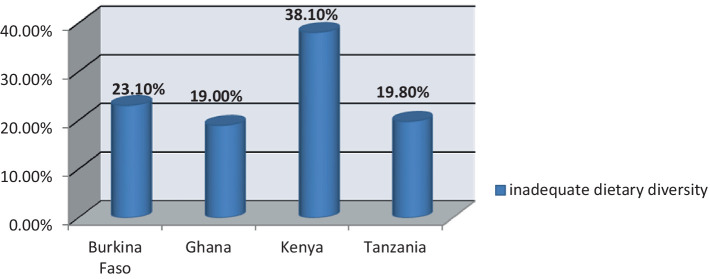
Prevalence of inadequate dietary diversity among pregnant women in four Sub-Saharan African countries.

The prevalence of inadequate dietary diversity among pregnant women in Burkina Faso, Ghana, Kenya, and Tanzania was 94.46% (95% CI: (94.30, 94.62)). The magnitude of urban and rural inadequate dietary diversity in four Sub-Saharan African countries was found to be 38.2 and 61.8%, respectively (Figure 2). Kenya (38.1%) and Ghana (18%) had the highest and lowest rates of inadequate dietary diversity among pregnant women, respectively (Figure 3).

### Measures of variation and model fitness

The null model’s results, which had a variance of 1.502113, demonstrated that there were notable variations in the inadequate dietary diversity throughout communities. About 31.4% of the total variation in inadequate dietary diversity in the null model occurred at the cluster level and can be attributed to community-level factors. Furthermore, the null model had the highest median odds ratio (MOR) value (3.20). This means that when individuals are randomly selected from one cluster at a higher risk of inadequate dietary diversity and another cluster at a lower risk, those at the higher risk cluster have 3.20 times higher odds of having inadequate dietary diversity than their counterparts. According to Model I’s intraclass correlation value, the differences between communities can be attributed to 20.98% of the variation in inadequate dietary diversity. Subsequently we created Model II using community-level variables and the null model. Based on the ICC value from Model II, cluster variations accounted for 20.43% of the variance in inadequate dietary diversity. In the final model (model III), both individual and community-level factors were responsible for about 70.95% of the variation in the likelihood of inadequate dietary diversity. The final model (model III), which had the lowest deviation (9,540.562) and the highest logliklihood ratio (−4770.281), was the best-fitted model. The model fitness was assessed using those parameters (Table 3).

**Table 3:** Model comparison and random effect analysis for inadequate dietary diversity among pregnant women in four Sub-Saharan African countries, DHS 2021–2022.

Parameter	Null model	Model I	Model II	Model III
Variance	1.502113	0.8734871	0.8485513	0.4364257
ICC	31.4%	20.98%	20.43%	11.71%
MOR	3.20	2.43	2.40	1.87
PCV	Reference	41.85%	43.51%	70.95%
Model fitness				
LLR	−15916.522	−5040.2241	−14834.828	−4770.281
Deviance	31,833.044	10,080.4482	29,669.656	9,540.562

ICC, interacluster correlation; LLR, logliklihood ratio; MOR, median odds ratio; PCV, proportional change in variance.

### Factors associated with inadequate dietary diversity among pregnant women in four Sub-Saharan African countries

Maternal education, distance to health facility, household wealth status, household media exposure, community ANC utilization, and reside in Burkina Faso were significantly associated with inadequate dietary diversity among pregnant women at a *p*-value of <0.05 in multivariable multilevel mixed-effect logistic regression analysis, where both the individual and community level factors were fitted simultaneously.

The odds of inadequate dietary diversity were 3.39 times higher among women who had no formal education compared to women who had secondary and higher educational levels (AOR = 3.39, 95% CI: 2.54, 4.54). The odds of inadequate dietary diversity were 1.36 times higher among women whose distance to health facilities was a big problem to access health services as compared to women whose distance to health facilities was not a big problem (AOR = 1.36, 95% CI: 1.16, 1.60). Inadequate dietary diversity was 2.97 times higher among women from poor wealth quantiles as compared to women from rich wealth quantiles (AOR = 2.97, 95% CI: 2.41, 3.65). The odds of inadequate dietary diversity were 1.84 times higher among women who had no media exposure compared to women who had household media exposure (AOR = 1.84, 95% CI: 1.45, 2.35).

The odds of inadequate dietary diversity were 1.21 times higher among women who had low community ANC utilization as compared to women who had high community ANC utilization (AOR = 1.21, 95% CI: 1.16, 1.60). Inadequate dietary diversity was 1.47 times higher among women from Burkina Faso as compared to women from Tanzania (AOR = 1.47, 95% CI: 1.09, 1.99; Table 4).

**Table 4:** Multivariable multilevel logistic regression analysis of individual-level and community level determinants of inadequate dietary diversity among pregnant women in four Sub-Saharan African countries, DHS 2021–2022.

Individual level variables	Model I AOR(95% CI)	Model II AOR(95% CI)	Model III AOR(95% CI)
Maternal age			
15–19	0.97(0.76, 1.23)		0.85(0.67, 1.09)
20–35	1		1
36–49	0.67(0.59, 0.77)		0.77(0.67, 1.88)
Maternal educational level			
No formal	7.92(6.00, 10.44)		**3.39(2.54, 4.54)**
Education	1.23(1.60, 4.01)		1.12(0.89, 2.89)
Primary	2.34(1.94, 2.83)		1.68(0.39, 2.03)
Secondary above	1		1
Husband educational level			
No formal	1.96(0.59, 2.41)		1.63(0.33, 1.99)
Education	1.61(0.29, 2.00)		1.42(0.14, 1.77)
Primary	1.47(0.21, 1.78)		1.36(0.13, 1.65)
Secondary above	1		1
Maternal occupation			
Not working	1.03(0.89, 1.19)		1.09(0.94, 1.27)
Working	1		1
Distance to health facility			
Big problem	1.34(1.14, 1.57)		**1.36(1.16, 1.60)**
Not a big problem	1		1
Number of ANC visits			
No visit	1.24(0.71, 2.17)		0.84(0.48, 1.48)
1–3	1.10(0.88, 1.37)		1.22(0.98, 1.52)
≥4	1		1
Total children ever born			
≤3	1		1
4–6	1.68(0.84, 3.37)		1.33(0.66, 2.66)
7–9	1.35(1.17, 1.56)		1.20(0.04, 1.38)
>9	2.11(1.55, 2.88)		1.74(0.86, 2.38)
Household wealth index			
Poor	2.17(1.81, 2.60)		**2.97(2.41, 3.65)**
Middle	1.49(1.26, 1.76)		1.77(0.49, 2.11)
Rich	1		1
Household media exposure			
No	2.05(1.62, 2.60)		**1.84(1.45, 2.35)**
Yes	1		**1**
Preceding birth interval			
≤24 months	0.96(0.81, 1.14)		1.03(0.87, 1.22)
>24 months	1		1
**Community level variables**			
Place of residence			
Rural		2.33(2.12, 2.55)	1.00(0.87, 1.17)
Urban		1	1
Community media exposure			
Low		1.45(1.23, 1.71)	1.64(0.36, 1.99)
High		1	1
Community poverty			
Low		1	1
High		1.32(1.15, 1.51)	1.12(0.95, 1.32)
Community women’s literacy			
Low		1.16(0.98, 1.37)	1.10(0.90, 1.33)
High		1	1
Community ANC utilization			
Low		1.34(1.18, 1.53)	**1.21(1.04, 1.41)**
High		1	1
Country			
Burkina Faso		1.40(1.15, 1.69)	**1.47(1.09, 1.99)**
Ghana		1.41(0.35, 0.47)	1.49(0.39, 0.62)
Kenya		1.14(0.13, 0.17)	1.17(0.14, 0.20)
Tanzania		1	1

Bold numbers indicate statistical significance.

## Discussion

Dietary diversity is crucial for pregnant women as it ensures they receive a wide range of essential nutrients necessary for their health and the development of the fetus (39). Inadequate dietary diversity during pregnancy can have significant consequences for both the mother and her fetus. It increases the risks of intrauterine growth restriction, abortion, low birth weight, preterm birth, prenatal and infant mortality, and morbidity (40).

This study aimed to assess the prevalence and determinants of inadequate dietary diversity among pregnant women in Burkina Faso, Ghana, Kenya, and Tanzania using 2021 and 2022 Demography and Health Survey data from each country. In this study, the magnitude of inadequate dietary diversity among pregnant women in four Sub-Saharan countries was 94.46% (95% CI: (94.30, 94.62)).

The prevalence of inadequate dietary diversity among pregnant women in this study was higher than the findings conducted in Ethiopia, 53% (41), 59% (42), 51.6% (43), Rwanda, 55.9% (44), Bangladesh, 55.5% (45). These discrepancies could be explained by variations in socio-economic conditions, cultural dietary practices, agricultural diversity, food security, and the effectiveness of public health interventions. In Ethiopia, for instance, studies have shown that factors such as food insecurity, family size, rural residence, lack of formal education, and absence of counseling about dietary diversity during antenatal care are significantly associated with inadequate dietary diversity. These factors could be less prevalent or more effectively managed in Ethiopia compared to the other countries mentioned (18, 42).

Moreover, each country has its unique set of challenges and policies that affect dietary diversity. For example, urbanization, economic status, and educational attainment have been identified as influencing dietary patterns in Burkina Faso. In contrast, Ethiopia’s efforts in improving dietary diversity might include different strategies, such as nutrition education and social safety programs, which could contribute to better outcomes (46).

In multivariable multilevel mixed-effect logistic regression analysis, where both the individual and community level factors were fitted simultaneously, maternal education, distance to health facility, household wealth status, household media exposure, community ANC utilization, and residence in Burkina Faso were significantly associated with inadequate dietary diversity among pregnant women at a *p*-value of <0.05.

The odds of inadequate dietary diversity were 3.39 times higher among women who had no formal education compared to women who had secondary and higher educational levels. It is in line with study findings in Ethiopia (6, 42, 47), Malawi (48), Zimbabwe (49), Nepal (50), and Brazil (51). This may be due to the fact that education increases awareness of the importance of a diverse diet, leading to better food choices, and educated women are more likely to be empowered to make decisions about their health and diet (50). Moreover, education can lead to better employment opportunities, providing the financial means to access a variety of foods and educated women are more likely to utilize healthcare services, including prenatal care, where they receive nutritional counseling.

The odds of inadequate dietary diversity were 1.36 times higher among women whose distance to health facilities was a big problem to access health services as compared to women whose distance to health facilities was not a big problem. This finding is consistent with previous findings in Ethiopia (42, 52), Tanzania (53), and Uganda (54). The possible explanation is that health facilities often provide education on the importance of a diverse diet during pregnancy. If these facilities are too far away, women may not receive this vital information. In addition longer distances can lead to fewer prenatal care visits, which means less opportunity for healthcare providers to assess and advise on nutritional status (18).

Inadequate dietary diversity was 2.97 times higher among pregnant women from poor wealth quantiles as compared to women from rich wealth quantiles. It is in line with the previous studies conducted in South Africa (55), Nigeria (56), China (57), and the United States (14). Poor wealth status is often associated with food insecurity, which can lead to a lack of consistent access to sufficient food for an active, healthy life. Economic constraints can also affect physical access to markets and stores where diverse foods are available, limiting the variety in one’s diet (50).

The odds of inadequate dietary diversity were 1.84 times higher among women who had no media exposure compared to women who had household media exposure. It is consistent with studies conducted in Ethiopia (50), Ghana (23), and Kenya (58), France (59). The possible explanation could be that media serves as a platform to disseminate information about the importance of dietary diversity for pregnant women, influencing their food choices. Through various media channels, pregnant women can learn about different types of foods and their nutritional values, which can encourage them to diversify their diet. Media can also raise awareness about available health and nutrition services, encouraging pregnant women to seek advice and support for maintaining a diverse diet (50).

The odds of inadequate dietary diversity were 1.21 times higher among women who had low community ANC utilization as compared to women who had high community ANC utilization. This is supported by the previous studies conducted in Ethiopia (60, 61), Somalia (61), Kenya (62), and Nigeria (63). The possible explanation might be that during ANC visits; women can receive personalized counseling on how to improve their diet, including information on the types of food to include for a balanced intake. Regular ANC visits allow for monitoring of the mother’s nutritional status and timely intervention if dietary diversity is found to be inadequate (53, 64). Inadequate dietary diversity was 1.47 times higher among women from Burkina Faso as compared to women from Tanzania. This suggests that various factors, possibly including socioeconomic conditions, cultural dietary practices, and access to a variety of foods, may contribute differently to the dietary habits of pregnant women in these two countries.

In Burkina Faso, only one in three women in urban areas reached the minimum dietary diversity. Factors such as household expenses, food security, and purchasing practices, like going to markets with a larger variety of choices, were associated with higher dietary diversity (65). In contrast, in Tanzania, more than one-third of pregnant women attending antenatal clinics in the coast region met the minimum dietary diversity. Factors like living near a health facility and having high nutrition knowledge were associated with adequate dietary diversity among pregnant women (53).

The use of recent large-sample national demographic and health surveys from four Sub-Saharan African countries was the strength of the study. This study’s use of mixed-effect multilevel logistic regression, which was not possible with standard logistic regression, to identify two-level factors (individual and community-level factors), was also the strength of this study. However, because several crucial variables, like maternal attitude and food security, were missing from the DHS data collection, the study was unable to incorporate these variables that might have been associated with the outcome variable. Furthermore, the absence of maternal nutrition-related data in several Sub-Saharan African DHS data sets restricts our potential to incorporate additional countries’ DHS data in this analysis.

## Conclusion and recommendation

This study concludes that inadequate dietary diversity among pregnant women is high. The study identified that both individual and community-level variables were determinants of inadequate dietary diversity. Therefore, the ministries of health in Burkina Faso, Kenya, Ghana, and Tanzania should give attention to those women who underutilize antenatal services, live in low wealth quantiles, and to women who did not get formal education opportunities while designing policies and strategies.

## Data availability statement

The datasets presented in this study can be found in online repositories. The names of the repository/repositories and accession number(s) can be found at: http://www.dhsprogram.com.

## Ethics statement

Ethical approval was not required for the studies involving humans because since this study is purely a secondary review of the DHS data, ethical approval is not needed. We enrolled with the DHS web archive, requested the dataset for our study, and were granted permission to view and download the data files. The studies were conducted in accordance with the local legislation and institutional requirements. Written informed consent for participation was not required from the participants or the participants’ legal guardians/next of kin in accordance with the national legislation and institutional requirements.

## Author contributions

AZ: Writing – original draft, Software, Methodology, Investigation, Formal analysis, Conceptualization. EM: Writing – review & editing, Validation, Methodology, Investigation. TT: Writing – original draft, Visualization, Resources, Methodology, Data curation, Conceptualization. BW: Writing – review & editing, Visualization, Supervision, Software, Funding acquisition, Formal analysis.
